# Teaching a difficult topic using a problem-based concept resembling a computer game: development and evaluation of an e-learning application for medical molecular genetics

**DOI:** 10.1186/s12909-019-1817-2

**Published:** 2019-10-24

**Authors:** Kamila Prochazkova, Petr Novotny, Miroslava Hancarova, Darina Prchalova, Zdenek Sedlacek

**Affiliations:** 1 Department of Biology and Medical Genetics, Charles University Second Faculty of Medicine and University Hospital Motol, V Uvalu 84, 150 06 Prague, Czech Republic; 2 Department of Teaching and Didactics of Biology, Charles University Faculty of Science, Prague, Czech Republic

**Keywords:** Medical genetics, Genomics, Bioinformatics, Medical databases, Interactive teaching application, E-learning, Problem-based learning, Gamification

## Abstract

**Background:**

Genetic testing rapidly penetrates into all medical specialties and medical students must acquire skills in this area. However, many of them consider it difficult. Furthermore, many find these topics less appealing and not connected to their future specialization in different fields of clinical medicine. Student-centred strategies such as problem-based learning, gamification and the use of real data can increase the appeal of a difficult topic such as genetic testing, a field at the crossroads of genetics, molecular biology and bioinformatics.

**Methods:**

We designed an electronic teaching application which students registered in the undergraduate Medical Biology course can access online. A study was carried out to assess the influence of implementation of the new method. We performed pretest/posttest evaluation and analyzed the results using the sign test with median values. We also collected students’ personal comments.

**Results:**

The newly developed interactive application simulates the process of molecular genetic diagnostics of a hereditary disorder in a family. Thirteen tasks guide students through clinical and laboratory steps needed to reach the final diagnosis. Genetics and genomics are fields strongly dependent on electronic databases and computer-based data analysis tools. The tasks employ publicly available internet bioinformatic resources used routinely in medical genetics departments worldwide. Authenticity is assured by the use of modified and de-identified clinical and laboratory data from real families analyzed in our previous research projects. Each task contains links to databases and data processing tools needed to solve the task, and an answer box. If the entered answer is correct, the system allows the user to proceed to the next task. The solving of consecutive tasks arranged into a single narrative resembles a computer game, making the concept appealing. There was a statistically significant improvement of knowledge and skills after the practical class, and most comments on the application were positive. A demo version is available at https://medbio.lf2.cuni.cz/demo_m/. Full version is available on request from the authors.

**Conclusions:**

Our concept proved to be appealing to the students and effective in teaching medical molecular genetics. It can be modified for training in the use of electronic information resources in other medical specialties.

## Background

Introduction to medical genetics is usually taught in the preclinical part of the undergraduate curriculum in the first and second year of study. Experience shows that especially medical molecular genetics, genomics and bioinformatics are considered difficult by many students. In addition, many find these topics less appealing and not connected to their future specialization in different fields of clinical medicine.

The use of genetic testing increases rapidly in all fields of medicine. Consequently, when current students finish their studies, it will be even more important and widespread. Furthermore, the role of clinicians, especially in the interpretation of the results of genetic tests, becomes crucial. Therefore, medical students must be well versed in molecular genetics and all aspects of genetic testing [1–4]. The responsibility of the teachers is to find a way to deliver to students the necessary knowledge and skills.

Our experience, as well as that of the others, shows that any clinical connection is welcome and ameliorates the learning process [5–7]. However, in the preclinical part of the study, the contact with clinical medicine is rather limited. Factors such as insufficient preparedness of the students, limited number of co-operative patients, the imperative of not disturbing the comfort of the patients significantly, and the often sensitive nature of genetic disorders limit broader involvement of patients in teaching. Practical laboratory work can enhance the learning process in a similar manner, but other hindrances including the cost of the reagents, access to instruments, demand for necessary skills, time requirements, incompatibility with teaching schedules, etc. also restrain its broader implementation.

A way to bypass these obstacles is to replace the direct involvement of patients and/or practical laboratory work at least partially by computer simulations, i.e. to use the in silico approach [8–10]. Authenticity is important for satisfaction of students with medical education [11]. This can be supported by the use of de-identified real data, provided that four attributes: anonymity, readability, consistency and medical correctness are met [12, 13]. The attractiveness of educational tools can also be enhanced by incorporating elements of gamification [14–16]. The employment of these approaches in teaching is especially appropriate in fields such as medical molecular genetics and genomics, disciplines strongly dependent on computers, electronic resources and electronic data analysis tools. However, nowadays this is increasingly true for most medical specialties, and the concept introduced here can be modified for virtually any field.

In this report we describe the development and evaluation of a new e-learning application for teaching medical molecular genetics which is based on the above mentioned principles. A demo version is available at https://medbio.lf2.cuni.cz/demo_m/ (Additional file 1). The full version is available on request from the authors.

## Methods

### Technical solution

We developed an electronic teaching application using the principles of problem-based learning and gamification to allow students to acquire general knowledge and skills through the solving of specific problems. The application is designed to run online as a website with access for students registered in the course. Most of the application is based on HTML (HyperText Markup Language) and JavaScript driven components. For validating the answers (a specific phrase or number) by the software as correct or incorrect, an asynchronous communication with back end written in PHP (Hypertext Preprocessor) is used. The application contains links to multiple internet resources, namely databases and online data analysis tools, which are necessary for successful completion of the tasks. Prior to each use the functionality of all links in the application must be checked by the authors and necessary updates must be made to keep the application functional.

### Participants

At our medical school, medical genetics is a part of the undergraduate course of Medical Biology in the first and second year of study. The practical class with the application takes place in the second year. About 200 students who were enrolled in our study took this practical class in November 2017, after the most recent upgrade of the application. It must be noted that this practical course was not the first time our students were exposed to medical molecular genetics; multiple preceding lectures and practical classes included this topic. However, in silico analyses and bioinformatics had not been covered in previous teachings.

### Effectiveness and feedback

A study was carried out to assess the effectiveness of the teaching application. We developed a test containing eight questions (six short-answer questions and two multiple-choice questions; Additional file 2). Students completed the test twice, one week before the practical class and then at its end (pretest and posttest). The posttest contained the same questions plus one additional box for personal comments. The tests were completed voluntarily and anonymously; students were only asked to use the same nickname in both tests. Short-answer questions were evaluated with 2 points - very good, 1 point - satisfactory, 0 point - unsatisfactory. In the multiple-choice questions Q1 and Q5 (Pedigree symbols and Mutation in electropherogram) only one answer was correct, and these questions were marked only as “correct” or “incorrect” (for the purpose of the evaluation of the whole test, correct and incorrect answers were assigned 2 and 0 points, respectively). To achieve consistency, all tests were graded by a single experienced teacher. In Questions Q3 and Q4 (Number of exons and Primer validation) some students proposed laboratory procedures but did not mention that the information could be found in databases or that the analysis could be done in silico. If the proposed procedure was principally correct, the answer was scored 1 point. The sign test with median values of the pretest and posttest was used to evaluate the results. The data were analysed using R 3.3.2 for Windows (R Foundation for Statistical Computing, R Core Team, Austria).

## Results

### Concept

**Fig. 1:**
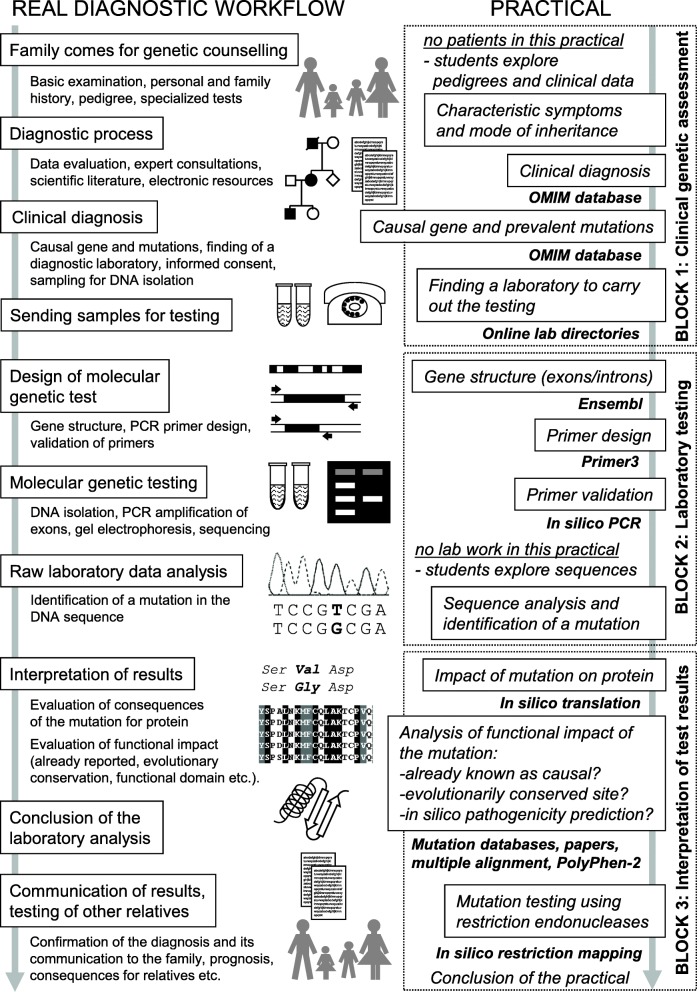
Overview of the real workflow of molecular genetic diagnostics and how the individual steps are reflected in the teaching application

The application provides students with a complex view of genetic testing for a monogenic disorder in a family. The real process is simulated as closely as possible (Fig. 1), and its individual steps are represented in the teaching application by a sequence of specific tasks. To complete the tasks, publicly available internet resources are employed in an identical manner to the daily routine of departments of medical genetics worldwide (Additional file 1). The databases and bioinformatic tools used are not intended to be memorized, but students should realize that many useful resources can be found on the internet, and that they can learn how to find and use them. Simultaneously, the application also deepens the knowledge of topics from previous lectures and practical classes such as modes of inheritance, gene structure, mutations, methods in molecular biology and many others, which are shown in the practical from a different perspective.

### Structure of the application

**Fig. 2:**
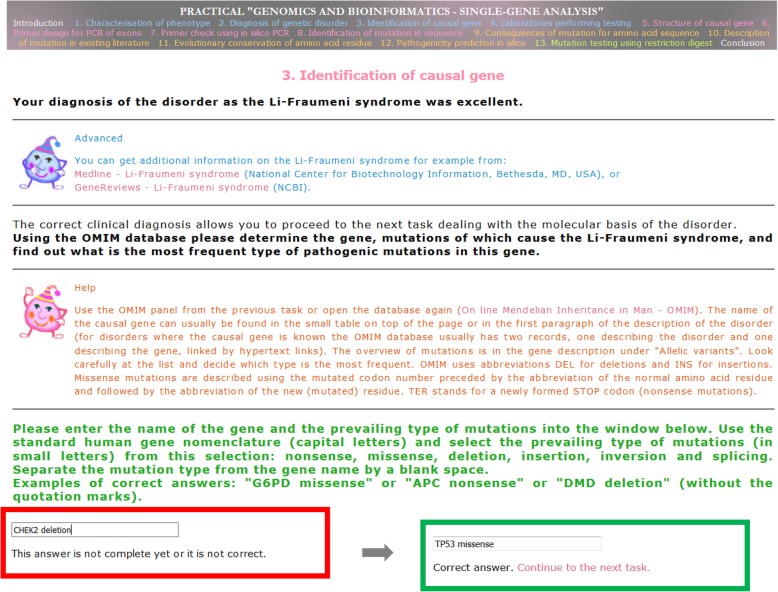
Example page of one task (identification of the causal gene and the prevailing type of mutations) from the teaching application. The alternative red and green frames at the bottom show how the system reacts to an incorrect or incomplete answer and a correct answer, respectively

Each web page presenting a task contains links to online databases and data processing tools needed to complete the task. The sequence of tasks follows the principle of levels in computer games. An answer box is at the bottom of each page, and if the answer is correct, the system allows the user to proceed to the next task. The pages also contain exhaustive “Help” sections specific to each task, “Advanced” sections presenting other web resources related to the tasks, and a few optional tasks. An example page with one task is shown in Fig. 2. The initial part of the application is identical for the whole class, but later the application branches and the students analyze one of six different families (with the same disorder but different pedigrees, symptoms, and mutations) to make the work more interesting and to prevent students sitting next to each other from completing exactly the same tasks. The application uses de-identified and modified clinical and laboratory data from families examined in our previous research projects.

In the practical class, individuals or teams of two students work under the guidance of the application, and the role of the teacher is merely supportive. Students vary in the time needed to complete the practical. The fastest ones need about 90 min, and all students finish in the allocated time (135 min). Students who have finished earlier are prompted to go back and explore in detail the “Advanced” sections and optional tasks. At the end of the practical, students can leave their comments in the system.

### Tasks

The application contains 13 tasks (plus four optional tasks) grouped into three blocks mimicking the real diagnostic process in a patient suspected of having a disorder. Currently the application uses one model disorder (an autosomal dominant cancer predisposition syndrome) but it can be modified or extended to any other monogenic hereditary disease. The comparison of the tasks in the application with the real diagnostic workflow is shown in Fig. 1, and the sequence of tasks with some tasks presented in detail (corresponding to the demo version of the application) is in Additional file 1.

Block 1 focuses on clinical assessment. Students explore pedigrees to determine the mode of inheritance of an unknown monogenic disorder, and phenotypes of affected family members to pick the most characteristic clinical features, allowing a successful search of a medical database. This search yields a provisional clinical diagnosis of the disorder, information on the gene associated with this disorder and on the prevailing type of mutations in that gene. The last step in Block 1 is a search for laboratories offering testing of this gene.

Block 2 simulates the laboratory analysis. Students do not perform any laboratory procedures in this practical (but they have trained them in the preceding practical classes). The application helps students realize that a substantial part of molecular genetic analyses is not performed in test tubes but in silico using various bioinformatic tools. Tasks in Block 2 include the analysis of gene structure of the causal gene (how many exons must be studied), primer design for PCR (polymerase chain reaction) amplification of exons, validation of the primers using in silico PCR and the analysis of an electropherogram from a capillary sequencer to identify the mutation.

Block 3 focuses on the interpretation of clinical relevance of the mutation. It starts with in silico translation to check whether the amino acid sequence of the resulting protein is affected. The clinical significance of the mutation is then assessed based on previous literature data, evolutionary conservation of the affected residue, and global impact prediction using an integrated in silico prediction tool. The last task, which is more freely linked to Block 3, explores whether restriction endonuclease digestion can be used to detect the specific variant, e.g. for quick analysis of other family members.

### Evaluation of effectiveness of the application

The impact of the application on the knowledge and skills of the students was assessed using pretest/posttest which contained eight questions (Additional file 2). In total, 185 students completed the pretest, and 169 students completed the posttest. The pretests and posttests were paired using the nicknames. In total, 137 tests could be unequivocally paired to allow exploring of individual improvements. Remaining tests could not be paired due to mismatches in the nicknames.

**Fig. 3:**
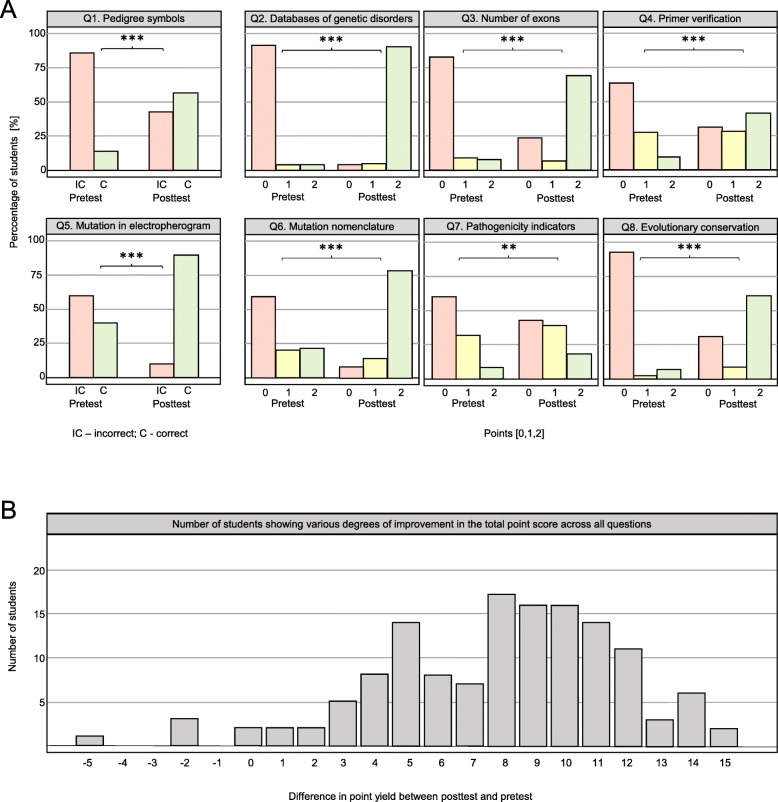
Comparison of the pretest and posttest results mapping the impact of the teaching application on students’ knowledge and skills. **a** Percentages of students obtaining each score (2 points - very good, 1 point - satisfactory, 0 points - unsatisfactory for short-answer questions (Q2-Q4 and Q6-Q8), or “correct” (2 points) and “incorrect” (0 points) evaluation for multiple-choice questions (Q1 and Q5)) in the paired pretests and posttests. *** and ** denote statistical significance at the level of *p* < 0.001 and *p* < 0.01, respectively. The results show overall improvement, although it varied between questions. **b** Number of students showing various degrees of improvement in the total point score across all questions (point yield for the whole test) between pretest and posttest. Numbers on the x-axis indicate differences in point yield between the two tests: negative and positive values indicate worsening and improvement, respectively, theoretically ranging between − 16 and + 16. The results clearly indicate substantial improvement for most students

We found a statistically significant improvement in the posttest results (*n* = 137, s = 121, *p* < 0.001) (Fig. 3). The data confirmed the hypothesis that students would in some cases answer correctly in the pretest but that there would be an increase in correct answers in the posttest. There was a statistically significant decrease of answers proposing laboratory procedures in Questions Q3 and Q4 (Number of exons and Primer validation): 43 in the pretest and only 6 in the posttest in the paired tests. This showed newly gained bioinformatic thinking and understanding that internet resources can yield answers faster and without the need for experiments.

Finally, we analyzed the comments collected in the posttests. In total, 127 (67.5%) students added a comment. Most of the comments were positive and some were useful in indicating possible problematic points in the application. Examples of comments from the posttests together with examples of comments collected during previous runs of the practical are in Additional file 3.

## Discussion

Our approach to teaching a rather difficult topic by solving of consecutive tasks arranged into a single narrative resembles a computer game. Authenticity is an important factor considered by students [11], and it is increased in our teaching application by the use of modified and de-identified real clinical and laboratory data. Another important factor is the quality of guidance through the activity [11]. Sections explaining background of the tasks and exhaustive help sections allow our students to proceed through the application without the direct involvement of the teacher, who is however present in the class to offer advice and help. These attributes make the practical attractive. Some individuals or teams proceed faster than others through the application, and the competition and joy of success enhance the teaching effect.

To our knowledge, no comparable concept aimed at medical genetics has been reported. Bioinformatics educational sessions during which the teacher introduced a problem, showed how to solve it using web resources, and students then completed the task on their computers have been described [8]. In another approach, virtual laboratory sessions followed the study of materials about laboratory and bioinformatic tools, and of specific cases [9]. During the sessions, students worked in groups of 15–16 under the guidance of a facilitator, and they proposed and discussed appropriate testing methods and bioinformatic resources to solve the case [9]. Both these approaches required strong involvement of teachers and assistants, and did not allow students to work entirely independently. A teaching system allowing students to work more independently has been presented, although with few details [10]. This system engaged bioinformatic tools in short tasks, but apparently lacked a central narrative. Nevertheless, it was found to significantly enhance learning and create positive attitudes toward bioinformatics [17].

We are aware that our assessment of effectiveness of the teaching application is limited by the absence of a comparison group of students who would receive teaching of the same topic in a different way (e.g. classical frontal instruction) [18]. However, it is difficult to organize the whole study as separate from the regular teaching process, and if a method is included as a part of the regular curriculum (and the topic is a part of the final examination), all students must be exposed to the same form of teaching to avoid discrimination.

In reflection on the feedback gained from the tests and comments, our teaching application is being continuously revised. Its modular structure allows for an easy addition of new tasks or upgrading of existing tasks. The current application deals with a monogenic disorder with a negligible genetic heterogeneity where the analysis of pedigrees and clinical data yields sufficient guidance as to which gene should be tested, and the students get familiar with principles of single-gene analysis. There has been a shift to whole-genome analyses recently, and we are finalizing another teaching application based on the same concept which illustrates the workflow of microarrays and exome sequencing.

## Conclusions

We developed an interactive application to enhance teaching of medical molecular genetics. The practical class also accesses topics from previous theoretical classes from a different perspective. Our concept proved to be appealing to the students and effective in teaching medical molecular genetics. It can also be modified and used for training in the use of electronic information resources in other medical specialties.

## Supplementary information

**Additional file 1.** The sequence of all tasks with three tasks shown in detail (corresponding to the demo version of the application).

**Additional file 2.** Pretest/posttest questions.

**Additional file 3.** Examples of students’ comments 1) from the posttest and 2) from previous runs of the application.

## Data Availability

Demo version of the application is available at https://medbio.lf2.cuni.cz/demo_m/. The full version is available from the authors on request. The datasets used and/or analyzed during the study are available from the authors on request.

## Abbreviations

HTML: HyperText Markup Language
PCR: polymerase chain reaction
PHP: Hypertext Preprocessor
