# Supplementary material for: Teaching a difficult topic using a problem-based concept resembling a computer game: development and evaluation of an e-learning application for medical molecular genetics
Source: BMC Med Educ. 2019 Oct 24;19:390. doi: 10.1186/s12909-019-1817-2 (PMC6813102; doi:10.1186/s12909-019-1817-2)
Supplement: Supplementary file 1 — Additional file 1. The sequence of all tasks with three tasks shown in detail (corresponding to the demo version of the application). [file 12909_2019_1817_MOESM1_ESM.pdf]

Additional file 1: The sequence of all tasks with three tasks shown in detail (corresponding to the demo version of the application)

PRACTICAL "GENOMICS AND BIOINFORMATICS - SINGLE-GENE ANALYSIS"

Introduction 1. Characterisation of phenotype 2. Diagnosis of genetic disorder 3. Identification of causal gene 4. Laboratories performing testing 5. Structure of causal gene 6. Primer design for PCR of exons 7. Primer check using in silico PCR 8. Identification of mutation in sequence 9. Consequences of mutation for amino acid sequence 10. Description of mutation in existing literature 11. Evolutionary conservation of amino acid residue 12. Pathogenicity prediction in silico 13. Mutation testing using restriction digest Conclusion

This is a Demo Version only. For the full version please contact the authors.

Introduction

Welcome to the practical **"Genomics and Bioinformatics"**! You will solve several tasks in clinical and molecular genetics using internet databases and online data analysis tools. You will learn about various internet resources for clinical genetics which you will use in your future professional practice. You will also get a basic orientation in resources used in molecular genetics which will help you to decide in requesting laboratory genetic tests for your patients and in the interpretation of results of these tests, as well as in the understanding of benefits and limitations of these methods in routine clinical use. The practical is based on real patient data and mutations identified in families analysed in research projects of our group. The data were anonymised and modified to make the families non-identifiable.

The overview of all tasks is in the banner on the top of your screen.

The tasks are grouped in three main blocks, each containing four tasks:

- **In the first block** you will find a necessary minimum for your orientation in internet resources for medical genetics, which must be understood by every clinician, such as searching in databases of hereditary disorders, genes and their mutations, and databases of laboratories offering molecular genetic testing.
- **In the second block** you will explore procedures and resources used in laboratories of medical molecular genetics, such as working with databases of nucleotide sequences and with tools for their online analysis, primer design and testing, and analysis of the output of an automated DNA sequencer.
- **In the third block** you will find analyses focused on the interpretation of laboratory findings, especially the analysis of mutations, their functional consequences and their pathogenicity and causality for the phenotypes observed in the family. The interpretation of results should be performed jointly by the laboratory and the clinician who requested the testing.

Apart is the last, 13th task, mutation testing in other relatives using restriction endonuclease digestion of PCR products from their genomic DNA.

We wish you to enjoy the exploration of internet resources for medical genetics.

K.Procházková, Z.Sedláček

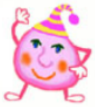

Help

Information marked by this picture and typed in this font colour should assist you in the practical work with the databases and tools. Please return to this section and read it carefully if you cannot find the answer in the database, if your online data analysis does not yield the right result, or if your answer is evaluated as incorrect.

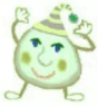

Optional task

Sections marked by this picture and typed in this font colour describe optional tasks. To complete them is not necessary for proceeding to the next task. These tasks extend your skills and may answer some questions which may arise while solving the compulsory tasks.

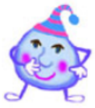

Advanced

Texts marked by this picture and typed in this font colour describe additional internet resources, which you can explore either right during your work on the compulsory tasks if you have enough time, or you can return to them after you successfully pass all compulsory tasks (from the "Conclusion" page at the end of the practical).
Please be aware that your answer will be evaluated as correct only if you use the main database or data analysis tool indicated in bold on the top of the page of the respective task. Answers obtained using alternative resources (listed in the "Advanced" section or found independently using web search) may not work for some tasks. While the translation of a nucleotide sequence into protein will always be the same, laboratories and their telephone numbers or suggested primer sequences can differ if you use different resources.

Continue

This is a Demo Version only. For the full version please contact the authors.

Not contained in the Demo Version: 1. Characterisation of phenotype

In this task students are shown eight pedigrees of families suffering from the same hereditary disorder with clinical information. Students select among several queries (lists of key words) one which best describes the mode of inheritance and the characteristic clinical symptoms of the disorder. The selected query is then used in OMIM search in the next task.

Example pedigree with phenotype information (y, age of tumour onset in years):

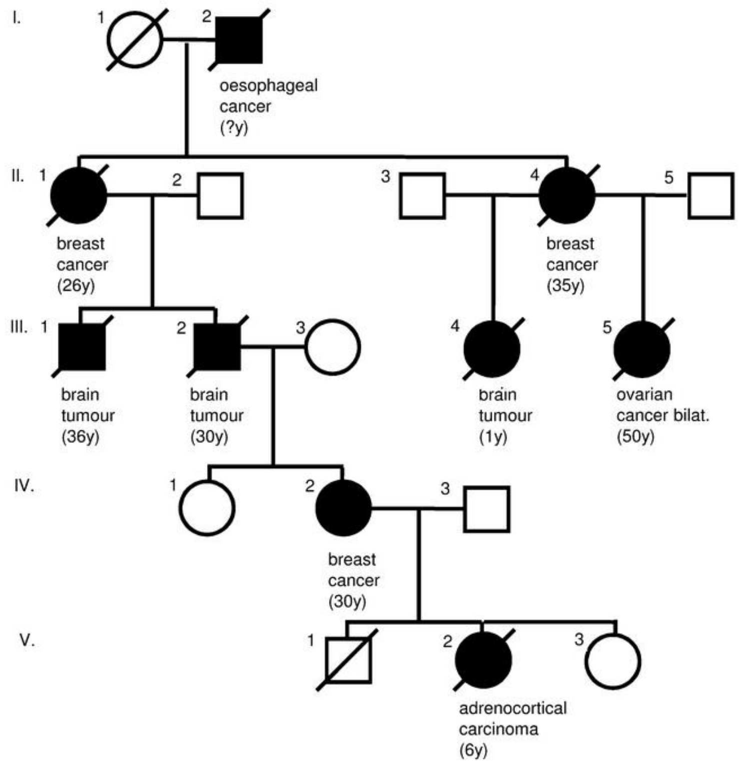

2. Diagnosis of genetic disorder

"adrenocortical carcinoma" AND "breast cancer" AND "soft tissue sarcoma" AND osteosarcoma AND "brain tumor" AND "autosomal dominant"

A very well formulated query.

If you enter this query into the search field of the Online Mendelian Inheritance in Man - OMIM database (Johns Hopkins University School of Medicine, Baltimore, MD, USA), the database will return relevant search results. As this query is specific enough, you will get only very few records. This will allow you to find the genetic disorder for which these symptoms are characteristic. You can read more details about the disorder in the OMIM database. **Please do not close the OMIM database because you will work with it also in the next task.**

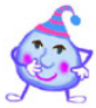

Advanced

Additional information on genetic disorders can also be retrieved from:
GeneReviews - search for any disorder
NCBI Genes and Diseases (both links at the National Center for Biotechnology Information, Bethesda, MD, USA).

Please enter the full name of the disorder (not only the abbreviation and excluding the 6-digit OMIM number) from the title of the OMIM item into the window below.
Examples of correct answers: "CYSTIC FIBROSIS" or "POLYCYSTIC KIDNEY DISEASE 1" or "DOWN SYNDROME" (without the quotation marks).

This is a Demo Version only. For the full version please contact the authors.

Your diagnosis of the disorder as the Li-Fraumeni syndrome was excellent.

Not contained in the Demo Version: 3. Identification of causal gene

In this task students identify the gene which is causal for the disorder they diagnosed clinically, and assess the most frequent type of mutations in this gene. Students use the OMIM database to obtain these answers. Similarly to all following tasks, students are also shown alternative resources containing the information in the "Advanced" section.

You have found that the Li-Fraumeni syndrome is caused by germline mutations in the TP53 gene, most of which are missense, and that these mutations affect different codons of the gene (different amino acid residues of the p53 protein) in different families.

Not contained in the Demo Version: 4. Laboratories performing testing

In this task students search the directory of laboratories performing testing of the TP53 gene and find the telephone number to the nearest laboratory. Students use the ORPHANET database to obtain the answer.

You have successfully found a laboratory which performs TP53 gene testing. For more frequent disorders many different labs exist, while for rarer diseases there are much fewer labs.

Summary of the first block of tasks

You have successfully completed the first block of four tasks focusing on the clinical genetics part of the work on our model case.

In these four tasks:

- you have identified the characteristic symptoms and the mode of inheritance of the disorder
- you have determined the clinical diagnosis of the disorder
- you have found which gene is causal for the disorder and what is the nature of mutations in this gene
- you have found laboratories performing the molecular genetic diagnostics of this disorder

At this stage the medical doctor who is not involved in laboratory tesing would pass the case to laboratory geneticists and he/she would return to it after obtaining the result from the laboratory. He/she would then participate in the interpretation of the finding, communicate the result to the family and decide about the follow-up care. In this practical you will also deal with the tasks performed by the laboratory. A doctor who is well oriented in the laboratory part of the examination will better understand the findings and the problems and limitations of laboratory analyses. In the following four tasks you will try, just in silico on your computer screen, to design the molecular genetic test and to perform testing in the affected family.

Continue

This is a Demo Version only. For the full version please contact the authors.

Not contained in the Demo Version: 5. Structure of causal gene

In this task students identify how many exons are contained in the main transcript of the TP53 gene. Students use the ENSEMBL database to obtain the answer.

Excellent, you have correctly determined that the main transcript of the TP53 gene has 11 exons.

After this task the practical branches and students analyse one of six families which differ in their pedigree structure, clinical features and, most importantly, in the genetic defect they carry. Students have a card at their desk indicating the number of the family they will explore (which is different from the families analysed by their neighbours). At the end of the practical students can return and analyse any of the remaining families if they are interested.

Not contained in the Demo Version: 6. Primer design for PCR of exons

In this task students design primers for amplification of a selected exon of the TP53 gene. Students use the Primer 3 tool to obtain the answer.

7. Primer check using in silico PCR

Congratulations to successful primer design. Before the primers are ordered they must be checked in silico once more using an independent tool if they indeed amplify just one DNA fragment of suitable length.

For example, the primers should not be located in repetitive sequences. Primer 3 allows to filter-out known human repetitive elements, but this function is not efficient for low-copy repeats. The primers should not amplify other regions of the human genome than the selected exon(s) of the TP53 gene.

Use the UCSC In-Silico PCR tool (University of California, Santa Cruz, CA, USA). If your primers are designed properly, just one DNA fragment will be amplified and the program will return just one in silico PCR product. The UCSC In-Silico PCR tool will also show you the chromosome region amplified. **Please use the primer sequences designed using Primer 3 in the previous task and enter them into the UCSC In-Silico PCR tool:**

```
OLIGO        tm    seq
LEFT PRIMER  59.87 CACTTGTGCCCTGACTTTCA
RIGHT PRIMER 60.95 AACCACCCTTAACCCCTCCT
```

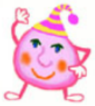

Help

Enter the primer sequences into the respective fields of the UCSC In-Silico PCR tool (left = forward, right = reverse primer). Change no default settings.

Check the result, especially whether there is just one PCR product, and what its length is.

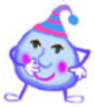

Advanced

Other options exist for in silico PCR, e.g.:
iPCR (Swiss Institute of Bioinformatics, Lausanne, Switzerland) (requires to enter a target sequence).
Repetitive sequences are the most serious problem in primer design. These can be identified and masked using RepeatMasker (Institute for Systems Biology, Seattle, WA, USA).

Please enter into the field below the length of the PCR product in base pairs.
Example of correct answer: "500" (without the quotation marks).

This is a Demo Version only. For the full version please contact the authors.

Congratulations, your primers are specific and give just one product of amplifiable length, and thus they can be used in PCR. The DNA fragment obtained using PCR must be purified and sequenced in two reactions using separately the left and the right primer.

Not contained in the Demo Version: 8. Identification of mutation in sequence

In this task students explore an electropherogram from an automatic Sanger sequencer which is shown on the page of the task together with the result of sequencing of a normal homozygote, and identify a position in the sequence trace where the patient has a heterozygous variant.

Yes, the position you have identified indeed represents a heterozygous point mutation. In a real experiment each variant has to be confirmed by sequencing of the reverse strand (using the other PCR primer) to clearly distinguish possible artefacts from real heterozygous positions. You compared the sequence of the patient with a sequence of a normal homozygote whose sequence is identical to the reference sequence of the human genome.

Summary of the second block of tasks

You have successfully completed the second block of four tasks focusing on the laboratory part of the work on our model case.

In these four tasks:

- you have determined the number of exons of the gene you are planning to analyse
- you have designed a pair of primers for PCR amplification of the selected exon (or two exons if they are located in vicinity)
- you have checked your primers using an in silico PCR tool if they indeed amplify only one fragment from the human genome
- you have performed PCR and sequencing of this fragment and identified a heterozygous position in the sequence curve, which could be the causal mutation in the family tested

However, it is still necessary to interpret the finding, i.e. to determine if the variant identified is indeed deleterious and responsible for the phenotype of your patient and his/her family. At the moment we do not know, for example, if the variant does not affect the third position of a codon and if it is not a synonymous or silent change, which does not lead to any change in the amino acid sequence. The causality of such a variant could not be a priori fully excluded, but the likelihood of causality would be much lower. Therefore it is necessary to explore the consequences of the nucleotide variant for protein function and to consider various effects the variant can cause. Very useful and often necessary is the participation of the clinician who requested the testing, who knows best the phenotype of the affected individuals from the family and who can best assess the similarity of their phenotype with phenotypes of previously published patients with the same or similar genetic defects. The following four tasks will deal with the interpretation of the finding you have obtained.

Continue

This is a Demo Version only. For the full version please contact the authors.

Not contained in the Demo Version: 9. Consequences of mutation for amino acid sequence

In this task students identify if and how the nucleotide variant affects the amino acid sequence of the protein produced from this allele. Students use the Six-Frame Translation tool to obtain the answer.

You have perfectly defined the mutation as NP_000537.3 (p53): p.(Ala138Pro) and you know it changes the amino acid sequence.

Not contained in the Demo Version: 10. Description of mutation in existing literature

In this task students explore a locus-specific mutation database to find out if the mutation they have identified has already been reported in the literature. Specifically, students use the Database of Germline TP53 Mutations to obtain the answer.

Your mutation has already been described in the literature. As you have found it was described in 1998.

Not contained in the Demo Version: 11. Evolutionary conservation of amino acid residue

In this task students assess the evolutionary conservation of the amino acid residue affected by the mutation. Students use the Clustal Omega tool to obtain the answer.

Yes, the evolutionary conservation of the amino acid residue affected by your mutation suggests that selection has not allowed changes in this amino acid position during evolution, and that this position can thus be functionally important.

12. Pathogenicity prediction in silico

The assessment of various parameters of the mutation (including evolutionary conservation of the position from the previous task), which can support its pathogenity, can be performed manually. However, **prediction programs exist which automatically integrate several of these parameters and yield a pathogenicity score estimating the functional impact of the variant.** In this task you will explore such an approach.

You will use the tool PolyPhen-2: prediction of functional effects of human nsSNPs (Harvard University). This program considers evolutionary conservation, known functional domains of the protein, spectra of amino acid residues in corresponding positions of related proteins, influence of the substitution on the protein structure and other parameters.

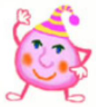

Help

Enter the protein identifier, e.g. "P53_HUMAN" (without the quotation marks; this is the identifier of the human p53 protein in the UniProt Knowledgebase). Leave the second field (sequence) empty. Enter the position (codon number) and the normal and "mutated" amino acid. (The selection is in one-letter amino acid code but if you move the mouse over the selection strips full names will appear; alternatively this table can also be used).
Then click on "Submit Query". You will have to wait for a while and press (even repeatedly) the "Refresh" button. When it appears, click the "View" link. Please open the HumVar algorithm (needs to be clicked on) which suits better for severe mutations in Mendelian disorders. The higher and closer to 1 the score is, the more pathogenic the variant is on the scale benign - possibly damaging - probably damaging.

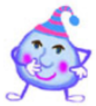

Advanced

Other prediction algorithms exist, e.g. SIFT (J. Craig Venter Institute Rockville, USA) or MutationTaster (Charité Berlin, Germany). If more prediction programs agree, the support for pathogenicity of the variant is stronger.

Please enter the pathogenicity score of your mutation calculated by the HumVar algorithm of PolyPhen-2 into the window below.
Example of correct answer: "0.975" (without the quotation marks).

This is a Demo Version only. For the full version please contact the authors.

Summary of the third block of tasks

The analysis using PolyPhen-2 also indicates that the mutation is likely to be highly deleterious.

You have successfully completed the third block of four tasks focusing on the interpretation of the molecular genetic finding.

In these four tasks:

- you have explored if the variant identified is exonic and if it changes the amino acid sequence, and if so, which amino acid position is affected and what is the new amino acid
- you have searched if this variant has already been described in the literature
- you have analysed if the amino acid position affected is evolutionarily conserved and thus likely functionally important
- you have performed in silico pathogenicity prediction

Most aspects considered in your analysis yielded evidence for pathogenicity of the variant identified in your family and for its likely causality for the phenotypes observed. This conclusion was made in collaboration of the laboratory with the physician requesting the testing. It confirms the diagnosis which was originally based only on the clinical picture and mode of inheritance. The identification of the causal mutation allows to modify the follow-up care for the patient tested. Concurrently, this finding also allows predictive testing of other relatives at risk as they may carry the same mutation. Today testing of other family members is performed using sequencing of the exon carrying the mutation. In the past, a cheap and fast diagnostics using restriction endonucleases was frequently used, which could perhaps be done even today in large pedigrees (which is usually not the case in the Li-Fraumeni syndrome). However, the approach is elegant (the mutation creates a new recognition site or destroys an existing site) and you will explore this method in the last task.

Continue

This is a Demo Version only. For the full version please contact the authors.

Not contained in the Demo Version: 13. Mutation testing using restriction digest

In this task students try to find out using an in silico restriction mapping tool if the mutation can be detected using restriction endonuclease digestion. Students use the Webcutter tool to obtain the answer.

Yes, the mutation can be detected e.g. using BalI, which has the recognition site TGGCCA, and if this sequence is mutated to TGCCCA, the site is destroyed. (Similarly it works for CfrI, which has a similar recognition sequence.) While the normal allele is cut in this position, the mutated allele is not. In addition to this site there is another sequence TGGCCA in the PCR product, and this second recognition site is present on both the normal and the mutated allele. If the sites are not too close this usually presents no problem, rather the opposite: the constant site can be used as a control of completeness of the digestion.

Conclusion

Congratulations, you have successfully passed all tasks of the practical Genomics and Bioinformatics.

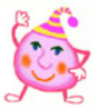

Help

Please let your teacher know.

In the three blocks of tasks you have performed:

- the clinical genetic analysis of a family and the clinical diagnosis of a hereditary disorder
- the molecular genetic analysis in an affected individual and the identification of a nucleotide variant in the gene responsible for the disorder
- the interpretation of the finding which you determined to be pathogenic and likely causal for the condition observed in the family

in addition you designed a test based on restriction endonuclease digest for targeted analysis of this mutation

We hope the practical has convinced you that internet offers many useful resources and analysis tools. You have also gained an idea about the course of analysis of a patient suspected to suffer from a hereditary disorder. We are convinced you will be alble to use this knowledge in the future, even if you are perhaps not directly involved in the field of medical genetics.

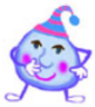

Advanced

Useful pages exist collecting multiple bioinformatic links. If you face a bioinformatic challenge, it is very likely that you will be able to find on the web suitable databases and/or tools to manage this challenge. You can explore these bioinformatics pages:
Introduction to Bioinformatics: online tools (Charles University Faculty of Science, Prague)
ExPASy Life Sciences Directory (Swiss Institute of Bioinformatics, Lausanne, Switzerland)
123 Genomics
Clinical Genetics Computer Resources (University of Kansas, Kansas City, USA)
The Sequence Manipulation Suite (University of Alberta, Edmonton, Canada)
and many others.

If you have time left you can return and explore the Advanced links from the previous tasks or analyse another family. The links below will open the respective pages in a new panel.

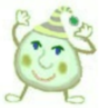

Optional task

Return to the previous tasks:
To the page with family selection: Family selection
To the beginning of the practical with pedigrees: Pedigrees

Not active in the Demo Version.

We would like to ask you for your comments on this practical and suggestions how it could be improved. We will be very obliged for your feedback. The form requires a name but you can use a nick.

Thank you.

K.Procházková, Z.Sedláček

Comments

Not active in the Demo Version.
