# Supplementary material for: Teaching a difficult topic using a problem-based concept resembling a computer game: development and evaluation of an e-learning application for medical molecular genetics
Source: BMC Med Educ. 2019 Oct 24;19:390. doi: 10.1186/s12909-019-1817-2 (PMC6813102; doi:10.1186/s12909-019-1817-2)
Supplement: Supplementary file 2 — Additional file 2. Pretest/posttest questions. [file 12909_2019_1817_MOESM2_ESM.pdf]

Additional file 2: Pretest/posttest questions

Please respond briefly. If you do not know the answer, leave the question unanswered. The completion of this form should not take more than 5 minutes.

Your name/nick

Q1. What does the last pedigree symbol on the right stand for?

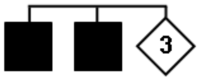

a) the third pregnancy ended in abortion
b) three consequent spontaneous abortions
c) the daughter (third in order) is unaffected
d) the third sibling of unspecified gender is unaffected
e) three younger siblings of unspecified gender are unaffected

---

Q2. Do you know any databases of genetic disorders? Please list their names.

Q3. How do you reveal the number of exons in the gene you decided to analyze?

Q4. How would you verify that the primer you designed does not bind to more loci in the genome?

Q5. A heterozygous missense mutation manifests in the sequence curve from Sanger sequencing as:

a) two neighboring peaks of normal height
b) two overlapping peaks of approximately half height
c) missing of one peak in the position of the mutation
d) beginning of a section of the sequence curve with many overlapping peaks
e) extending the length of the sequence curve by one peak in the position of the mutation

---

Q6. What does the expression p.(Gly480Cys) mean in molecular genetics?

Q7. What parameters or attributes of a genetic variant can give evidence for its negative influence on the expression of a gene and on its causality for the patient´s affection?

Q8. What can a medical geneticist infer from multiple alignment of amino acid sequences of the protein studied from several animal species?

We ask you for your brief comment on the e-learning application. *(field not contained in pretest)*

Thank you for cooperation.
