# Supplementary material for: Teaching a difficult topic using a problem-based concept resembling a computer game: development and evaluation of an e-learning application for medical molecular genetics
Source: BMC Med Educ. 2019 Oct 24;19:390. doi: 10.1186/s12909-019-1817-2 (PMC6813102; doi:10.1186/s12909-019-1817-2)
Supplement: Supplementary file 3 — Additional file 3. Examples of students’ comments 1) from the posttest and 2) from previous runs of the application. [file 12909_2019_1817_MOESM3_ESM.pdf]

Additional file 3: Examples of students' comments

1) from the posttest

Appreciating the structure and aims of the teaching application

We appreciate the work put into setting up this practical. It was very instructive and informative.

A very helpful, fun and beautiful way of teaching molecular genetics.

It was very insightful and I appreciate this new and improved approach to learning. Thank you.

Appreciating the benefit for future medical practice of the students

Thank you very much, it was great, we appreciate it strongly. We enjoyed it perfectly! Thank you that you teach us that for real understanding the topic, not only for the teaching process itself. Thank you!

I liked this practical, it was illustrative and I will better remember the topic.

Pointing to some problems with the structure of the application

The practical was interesting but I think I would not reproduce it again without the instructions; I was lost a little.

The practical was interesting but I am afraid that the clinical application would not be 100 %.

To my opinion too much routine work and copying, the effort to teach students the names of webpages which would not be remembered even until the next practical.

Pointing to some problems with the instructions in the application

Very nice and diversified; question and explanation for task 11 too vague.

Interesting practical, definitely. I had some problems with the last 3 tasks, it was not fully clear from the "Help" text.

Some tasks can be completely understood using "Help" texts but in some of them some details were missing – e.g. which of the results should we choose when the program proposes 7 different results or that the program automatically shows also the results of the preceding study group.

2) from previous runs of the application

Appreciating the structure and aims of the teaching application

Really enjoyed this program. It was a great tool to illustrate the practical application of lecture topics.

The practical was very informative and interesting. It showed us a more practical approach to the more theoretical aspects we have discussed in class.

Clear, beautiful and logical.

Very nice to apply theoretical knowledge in rather practical tasks. Giving an opportunity to see, why it´s important to be informed concerning genetics.

Provided insight into how a diagnosis can be made, starting at the family tree. Allowed a glimpse into the work of a geneticist and outlined the most important internet resources. It was also a refreshing change in the normal structure of the practical.

I think these practical tasks are the best way for students to learn how genetics work in practice, with up to date sites and technology. We should have more classes like these.

Appreciating the benefit for future medical practice of the students

Excellent program for summary, revision and consolidation of our knowledge acquired from previous lectures and practicals. It´s good to prove that what we learn in theory is really applicable in true situations. Thanks for this practical and hat off for the authors!

This task was very informative and we learned a lot which we can use in our practice at the hospital. I hope the students that come after us will use this tool a lot more, because it is extremely interesting and useful. Thanks for now.

Well prepared and coherent practical. I would particularly appreciate the connection with practice and also the interactive familiarization with internet portals we can use. Certainly, this form (when the student himself searches for the information, well guided by "Help" information) is more effective than the situation when internet resources are only one by one presented by teacher. Thank you!

This Medical Biology practical was great once it was very different from what we normally have and made me see how directly and effective genetics can be.

Pointing to some problems with the structure of the application

This was interesting, but a bit difficult. It was a lot of information to read.

So, quite demanding for the morning!

Generally funny… only a little slow computer… but nothing terrible. Respect for creation of such a practical.

Very nice, the answer fields could accept more answers (for not to be confused by wrong answer due to capital/lowercase letters). It´s a pity that it´s possible to go through even without a deeper understanding. But for fair students is this really interesting.

Quite interesting pratical...but sometimes a little mechanical. PCR song rules!!

I am so awesome and you are not. My work here was very good and fabulous and I hate molecular biology. It is a madness.

Never more!

Pointing to some problems with the instructions in the application

Excellent fresh practical which passed unbelievable quickly. Not-working Harvard database was an unpleasant interruption.

The only problem are non-cooperative and non-functional databases, I am satisfied from my view.

Very well prepared practical. It was fine to try it on. Thanks. "Help" texts were a little too simplified but it´s probably the prevention of a huge amount of questions.

Nice and thrilling story! Really interesting and good, how the links to databases etc. are prepared. Maybe, it would be useful to refine the instructions for answer fields.

Thank you, very interesting. Only from the task "primer design" I didn´t catch complete context and I was rescued by mechanical "Help" only.
